# Machine learning for detecting DNA attachment on SPR biosensor

**DOI:** 10.1038/s41598-023-29395-1

**Published:** 2023-03-06

**Authors:** Himadri Shekhar Mondal, Khandaker Asif Ahmed, Nick Birbilis, Md Zakir Hossain

**Affiliations:** 1 ANU College of Engineering, Computing and Cybernetics, The Australian National University, Canberra, ACT 2600 Australia; 2 Biological Data Science Institute, The Australian National University, Canberra, ACT 2600 Australia; 3 Data61, Commonwealth Scientific and Industrial Research Organisation (CSIRO), Canberra, ACT 2601 Australia; 4 Australian Centre for Disease Preparedness (ACDP), CSIRO, Geelong, VIC 3220 Australia; 5 Faculty of Science, Engineering and Built Environment, Deakin University, Burwood, VIC 3125 Australia; 6 Faculty of Science and Engineering, Curtin University, Perth, WA 6102 Australia

**Keywords:** Computational biology and bioinformatics, Engineering

## Abstract

Optoelectric biosensors measure the conformational changes of biomolecules and their molecular interactions, allowing researchers to use them in different biomedical diagnostics and analysis activities. Among different biosensors, surface plasmon resonance (SPR)-based biosensors utilize label-free and gold-based plasmonic principles with high precision and accuracy, allowing these gold-based biosensors as one of the preferred methods. The dataset generated from these biosensors are being used in different machine learning (ML) models for disease diagnosis and prognosis, but there is a scarcity of models to develop or assess the accuracy of SPR-based biosensors and ensure a reliable dataset for downstream model development. Current study proposed innovative ML-based DNA detection and classification models from the reflective light angles on different gold surfaces of biosensors and associated properties. We have conducted several statistical analyses and different visualization techniques to evaluate the SPR-based dataset and applied t-SNE feature extraction and min-max normalization to differentiate classifiers of low-variances. We experimented with several ML classifiers, namely support vector machine (SVM), decision tree (DT), multi-layer perceptron (MLP), k-nearest neighbors (KNN), logistic regression (LR) and random forest (RF) and evaluated our findings in terms of different evaluation metrics. Our analysis showed the best accuracy of 0.94 by RF, DT and KNN for DNA classification and 0.96 by RF and KNN for DNA detection tasks. Considering area under the receiver operating characteristic curve (AUC) (0.97), precision (0.96) and F1-score (0.97), we found RF performed best for both tasks. Our research shows the potentiality of ML models in the field of biosensor development, which can be expanded to develop novel disease diagnosis and prognosis tools in the future.

## Introduction

Optical biosensors are getting popularity in multi-dimensional research, ranging from fundamental biological and bio-medical research, to environmental and agricultural monitoring programs^1^. Surface plasmon resonance (SPR)-based optical biosensors are clearly widely used biosensors, due to their affordability and precise binding affinity across biomolecules, allowing high-throughput results for downstream analysis^2^. SPR biosensors are widely being used in analyzing bio-molecules (e.g. proteins, antibodies, nucleic acids and enzymes) and their molecular interactions^3^.

The study of bio-molecules, especially, DNA, is directly linked over 400 diseases diagnosis and several diagnostic characterisation techniques has been developed based on DNA detection and their binding properties^4^. e.g. diagnose pathogens in bacteremia patients^5^, leishmaniasis (skin diseases)^6^, chronic pulmonary aspergillosis^7^, urinary tract infections^8^ etc. Among different bindings, DNA hybridization is a significant biological procedure, where two single-stranded, complementary DNA bind together, by forming a double-helix DNA. Due to the ability to detect DNA hybridization in real-time and without any labeling, SPR-based biosensors have been deployed in detecting infectious components or associated DNA-mutations, resulting in hepatitis-B complex, cancer, and numerous congenital disorders^9^.

While most of these detection and diagnosis works involve biomedical studies, it becomes hectic to work with large datasets, with numerous dependent and independent variables to work on. Integrating machine learning (ML) models will not only leverage the disease diagnosis procedure but also be helpful to develop precise biosensors, designing personalized medications and predicting disease prognostication for susceptible individuals. ML-models are being widely used for predicting numerous diseases, e.g. COVID-19^10^, autism spectrum disorder^11^, cancer^12^, multiple sclerosis^13^, diabetes^14^ and mental health^15^. Vitor and Cleber^16^ developed an ML model to predict COVID-19 patients’ stay at special care facilities, based on physiological features resulting in a decision system, which showed potential to be applied in several different diseases, with low processing requirements. While most of these studies utilize patients’ physical and physiological data, derived from different biosensors, there are only a few handful studies, focused on developing a highly accurate biosensor. The probable reason can be the unavailability of a large and authentic dataset for developing and testing models with high confidence.

For our study, we found a dataset^17^, used to develop SPR-based biosensors, suitable to develop an ML model for DNA detection and classification. The dataset consists of reflective angles of 632.8 nm light at different thicknesses of gold and DNA-attachment stages, with their corresponding permittivity and permeability scores. The hypothesis we were tested involves, *is it possible to detect the presence and absence of DNA (DNA detection) from different reflective angles and associated independent variables and extend the model further to distinguish single and double-stranded DNA (DNA classification)?* Generally, according to the law of light, a line perpendicular is drawn between the incident and reflective lights^18^, and the angles drawn between them are called incident and reflective angles. In the case of SPR, incident light is applied on various surfaces at definite angles and intervals (for example, 40$^{0}$–89$^{0}$ at interval of 0.05$^{0}$) and the resulting reflective angle is captured at real-time to interpret the properties of the surface.

To test the hypothesis, we have evaluated numerous ML models, namely, random-forest (RF), support vector machine (SVM), k-nearest neighbor (KNN), decision tree (DT), logistic regression (LR) and multilayer perceptron (MLP), and proposed the best-performing model for both tasks. The RF classifier is based on a decent number of decision trees constructed by random data selection^19^. A group of theoretically potent machine learning algorithms is grouped under the name SVM. SVM has a number of benefits such as dealing with small sample regime, low dimensional data etc., in addition to being the most popular and reliable classifier for categorization^20^. The DT is a supervised technique, where the nodes in a decision tree stand in for attributes, the branches for decisions, and the leaves for labels for each instance. Another supervised learning technique called KNN, which develops model based on n-dimensional trained data^21^. MLP is unlike to SVM or naive bayes classifier but performs slightly differently. Discrete set observation is done by LR generally, mainly used for producing probability value^22^.

Even though, there are numerous studies on DNA detection^23,24^ and disease severity predictions as stated above, to best of our knowledge this current study, in our knowledge, is the first study which explored SPR-based dataset to detect and classify DNA—which open a new horizon at the field of biosensor development. The major contribution of the current study is to explore reflective angles on different gold surfaces of biosensors and utilize these parameters to classify DNA detection and classification tasks. While it is often a laborious and costly job to produce reflective angles for different surfaces and deposited biomolecules, current study set-up a baseline to develop innovative ML-based models for SPR-based bio-sensors, testing and predicting their performances at different parameter regimes. Our model for classifying and detecting DNA will ensure development of highly accurate biosensors, and assist in developing accurate classifiers for disease diagnosis and prognosis. Furthermore, current study wish to contribute to the tremendously expanding SPR-based dataset and relevant techniques, which needs investment from biosensor development communities.

## Methodology

### Data set and pre-processing

**Figure 1:**
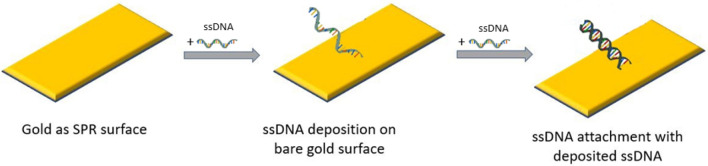
A schematic diagram of DNA attachment on gold surface of SPR-biosensor.

The dataset, utilized in the present study is collected from^17^. The dataset was generated through SPR technology which is presented in Fig. 1. In the biosensor surfaces, gold plates of varying thickness were used and reflective angles of 632.8 nm light were measured. Different permittivity and permeability values were also included within the measurements. The overall dataset was collected at three different stages of DNA binding on Gold plates, 10,000 datapoint for each, namely—bare, immobilized, and hybridization stages. The bare stage was considered when there was no DNA on the gold surface. The immobilized stage was considered when a single-stranded DNA (ssDNA) deposited on the top of the gold layer. The hybridization stage was considered when the deposited ssDNA attach with another ssDNA and form a double-stranded DNA (dsDNA) molecule. We divided the dataset into two sub-dataset for two different models. For DNA detection model, we combined data-point from immobilized and hybridization stages into “DNA presence”, whereas data point from the bare stage goes under “DNA absence”. For the classification model, we kept all stages the same as the original.

We have done a few ablation studies to analyze the dataset for model development. We explored the distribution of gold thickness using a tree map and analyzed the variances of gold thickness with average reflective angles, in terms of the different dataset. The ablation study was helpful to detect the low variance within each data point and hyper-parameter optimisation of the models. Finally, a min-max normalization technique was applied for data normalization before applying feature selection approach.

### Feature selection

Several feature extraction techniques^25^ can be used, e.g. statistical dependency (SD), minimal-redundancy-maximum-relevance (MRMR), random subset feature selection (RSFS), sequential forward selection (SFS), SFFS (Sequential Floating Forward Selection) etc.^26^. The non-linear dimensionality reduction approach known as t-SNE is frequently used to visualize large datasets. Natural language processing (NLP)^27,28^, speech processing^29,30^ are a few of the key uses of t-SNE. In t-SNE feature extraction, Kullback–Leiber (KL) divergence is used to assess divergence within classifiers, followed by gradient descent to minimize the KL divergence. In current study, t-SNE was implemented for clustering the data according to their different stages or conditions.

### Evaluation of different ML models

**Figure 2:**
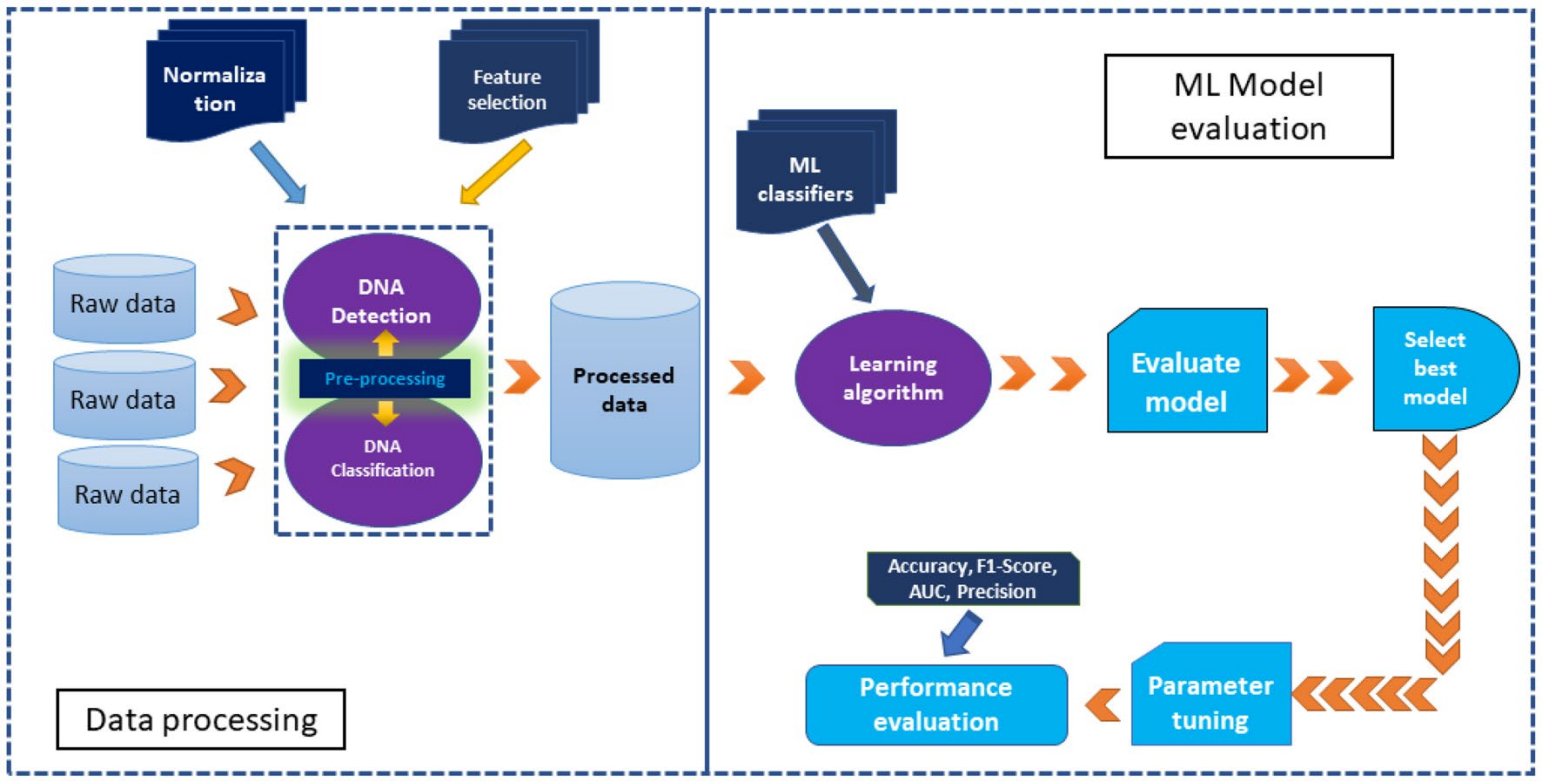
A flowchart of present study.

After feature selection, we applied several ML models individually for each DNA detection and classification model. A flowchart of the work is presented in Fig. 2. This ML model development is divided into two stages named data processing and ML model evaluation. At first, raw data was pre-processed separately for two different models. DNA detection model address 2-class classification problem, focusing on presence or absence of DNA on gold surface; while DNA classification model predicts different types of DNA on gold plate surface. After pre-processing with min-max normalisation and t-SNE based feature selection approach, several ML models were applied for both tasks. All parameters and hyper-parameters of different models were kept in default settings. Based on the evaluation matrices, the best fitted model was identified for downstream hyper-parameter optimization steps.

### Evaluation matrices

Model’s performance is usually measured using classifier’s performance indicators. Accuracy is the key findings, measuring the performance of a model which is the exact to total prediction. Precision and recall are also important parameters. Precision indicates actual positive outcome while recall indicates part of accurate prediction among the positive outcomes.

Current research used accuracy, F1-score, AUC and precision scores to compare performances for different models. The area under the curve (AUC), which depends on sensitivity, is provided by the receiver operating characteristic (ROC) curve, which also provides a broad overview of the model. An improved classifier indicates by higher AUC value. We have conducted 10-fold cross-validation and calculated standard error which calculates standard deviation from average scores for a specific evaluation matrix. To visualise, a confusion matrix was constructed to show the outcomes of a classifier. As a result, the possibility of true-negative (TN), true-positive (TP), false-negative (FN), and false-positive (FP) outcomes can be extracted. For both DNA detection and classification model, confusion matrix was also constructed to visualise the performances.

### Hyperparameter optimization

RF showed best performing model among all models. So, tuning of RF model was performed. As RF is a meta estimator, it utilizes averaging to increase predicted accuracy and reduce overfitting after fitting numerous decision tree classifiers to distinct dataset subsamples. Sklearn library’s RF model comprises 19 hyper-parameters. The most important hyper-parameters are n-estimators, max depth, max features and max samples. We have conducted experiments with different combinations of hyper-parameters, and found max-depth has significant effect on model performances and we optimised it for the best performance.

## Results

### Data distribution and feature extraction

**Figure 3:**
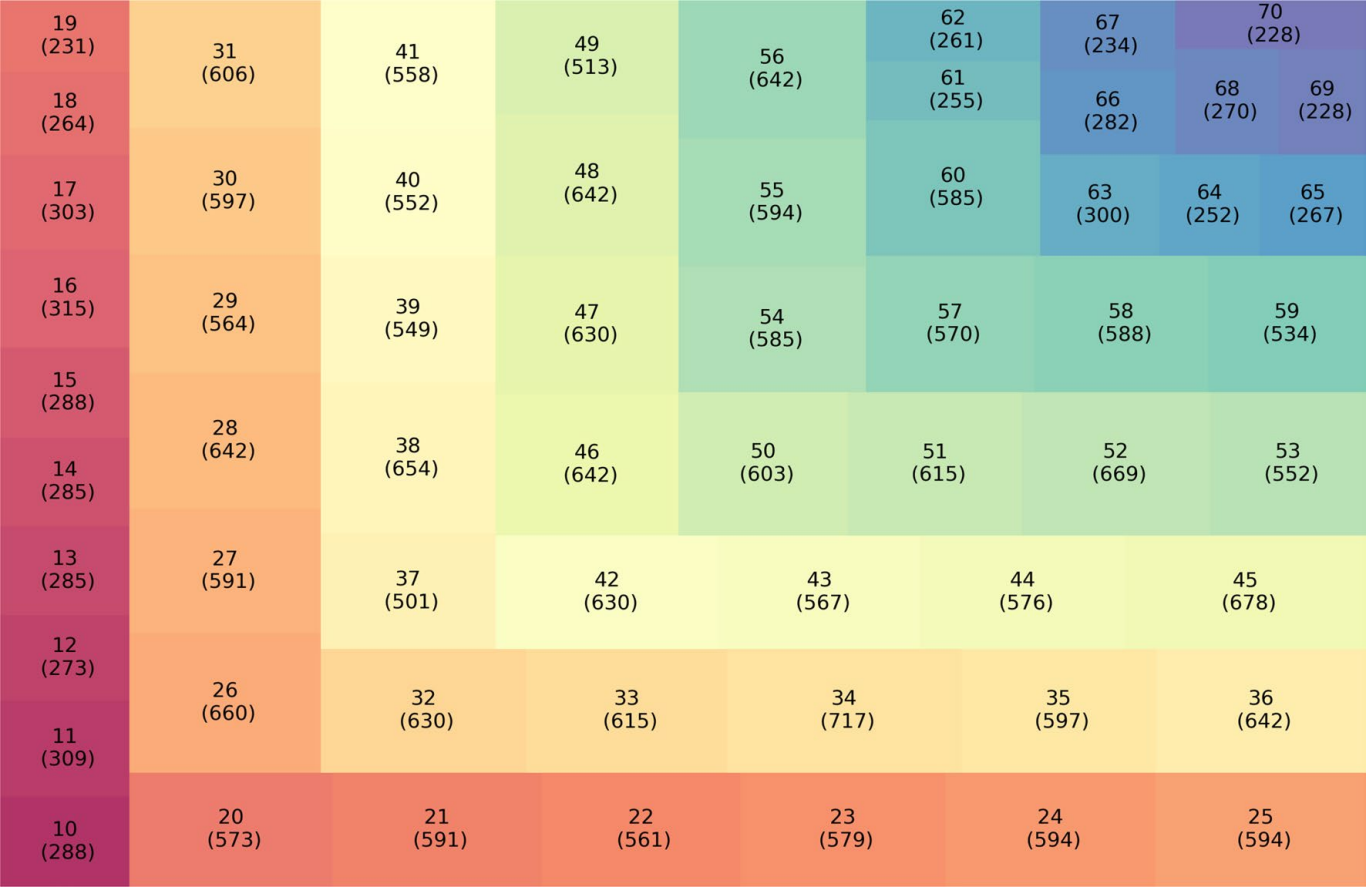
A treemap of gold thickness and the corresponding number of counts of reflective angles.

The overall SPR dataset was subjected to exploratory statistical analysis. As permittivity, permeability and gold thickness play significant roles in SPR sensing, these parameters were taken into consideration and characterizations were done for evaluating SPR performance. The gold thickness of SPR biosensors plays an important role in reflective index measurements. In order to closely inspect the data, we have constructed a treemap in Fig. 3, where the values inside boxes and brackets represent the thickness of the gold layer and the number of counts of reflective angles appeared at that specific thickness respectively. For example, 31 (606) indicates, at 31 mm thickness of gold, 606 reflective angles had been reported. We found gold thickness distribute from 10 to 70 nm, and within the range of 31 to 59 nm, the reflective angle counts are over 500. The top two counts of 717 and 669, were found in biosensors with gold thicknesses of 34 and 52 nm respectively. On the other hand, the least count of 228 was observed in 69 and 70 nm gold.

**Figure 4:**
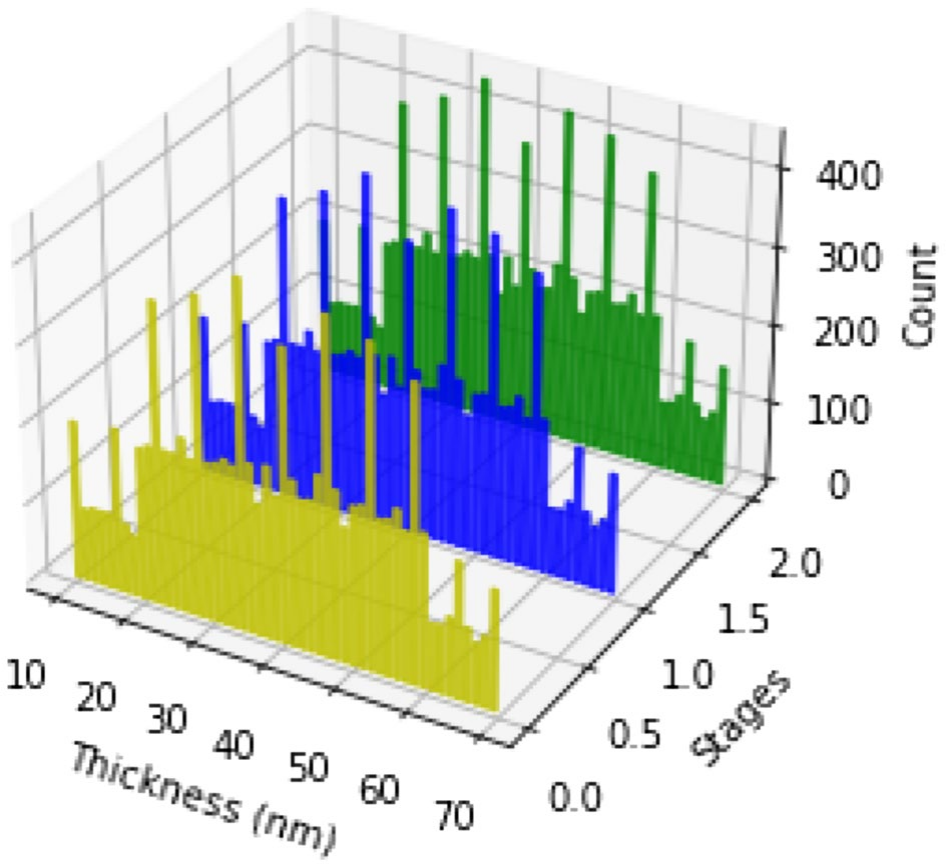
A histogram of counts of reflective indices at different gold thicknesses and DNA deposition stages. 0, 1, and 2 represent bare, immobilization, and hybridization stages.

Further, we explored the gold thickness distribution at different DNA-deposition stages. From Fig. 4, we observed similar bell-shaped gaussian distributions of gold thickness across different deposition stages. The histogram shows reflective angle counts were higher at 20-60 mm thickness of gold surface, which highest and lowest peaks were over 400 and just below 100 mm. The number of counts of reflective angles at different stages and thicknesses are presented in Y-axis. There is a small reflective index variance among bare, immobilization, and hybridization stages.

**Figure 5:**
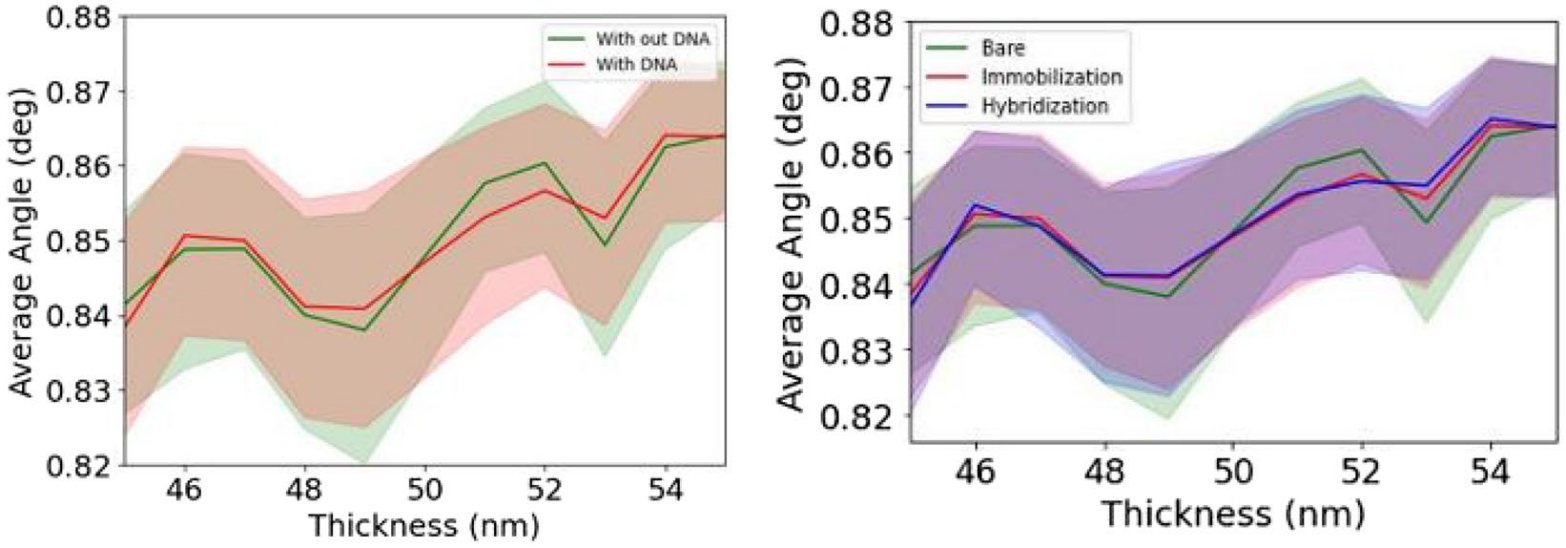
Gold thickness variances at different average angles for DNA detection (left) and classification (right) tasks.

**Figure 6:**
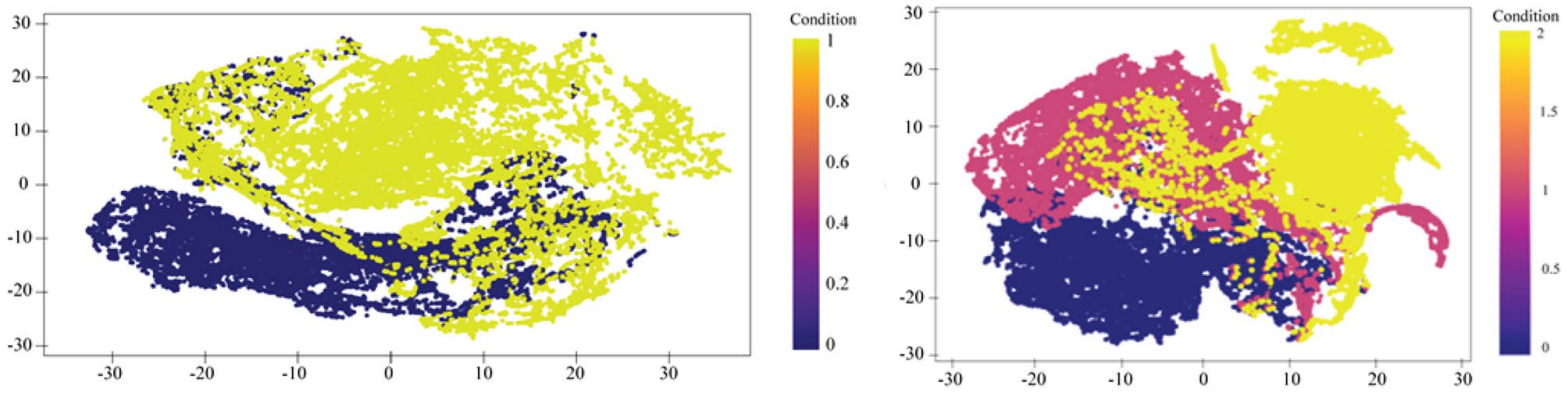
tSNE-based data distribution for DNA detection (left) and classification (right) tasks.

To address the problem of low-variance, we strategically sub-divided our data for two different models, namely DNA detection and classification models and observed the average reflective angle differences for both dataset. From Figs. 3 and 4, it is evident that, there are numerous counts of reflective angles at specific gold thicknesses. In terms of exploring angle values, we systematically reduced the angle data by taking the average of reflective angles at specific gold thickness and stage. The Fig. 5 indicates low-variances in average reflective angles at different gold thicknesses. The variance become lesser for DNA detection model, compared to DNA classification model. For better performance, we further utilised t-SNE net based feature extraction method to extract features. Feature extraction was done for extracting and reducing the dimension, and the clusters for different models are presented in Fig. 6. It can be seen that, after applying t-SNE, it is possible to differentiate the data-points for both detection and classification models, but still some data overlaps, which can interfere model’s performance.

### Performances of different models

We have utilised six-different supervised ML models and found RF and KNN achieved the best accuracy scores for both detection and classification models. For every model classes, the performances for DNA classification models were higher than that of DNA classification models. The highest accuracy 0.94 for DNA classification model was observed consistently across RF, DT and KNN models, while LR provides only 0.81 accuracy. the highest accuracy of 0.96 for DNA detection model was observed in both RF and KNN, while slightly low accuracy of 0.95 observed in DT and SVM, followed by 0.93 for MLP and 0.84 in LR. It is evident that, with 10-fold cross-validation, we have found a very low standard deviation (below 0.0001) for each model.

In terms of F1-score and precision scores (see Table 1), DNA detection models outperformed compared to detection models. In the case of precision score RF, DT and KNN showed 0.96 for detecting DNA on the SPR surface whereas lower score of predicting surface DNA of 0.86 was resulted by LR. The highest precision score of 0.95 achieved by RF, KNN and DT for hybridization. For immobilization same classifiers achieved 0.92. For F1 score, DNA detection on SPR surface shows 0.97 for RF and KNN, while LR got 0.89, performing lowest score. For immobilization 0.93 score was resulted by RF while LR achieved only 0.77, resulting lowest. For hybridization RF performs best as 0.95 while LR was the lowest with 0.82.

**Table 1:** Performance comparison of DNA detection and DNA classification.

DNA detection model			DNA classification model		
	Absence	Presence	Bare	Immobilization	Hybridization
DT					
Precision	0.93	0.96	0.95	0.92	0.95
F1-Score	0.93	0.96	0.95	0.92	0.94
SVM					
Precision	0.98	0.92	0.93	0.87	0.88
F1-Score	0.90	0.95	0.91	0.88	0.88
KNN					
Precision	0.97	0.96	0.95	0.92	0.95
F1-Score	0.94	0.97	0.95	0.92	0.95
MLP					
Precision	0.95	0.93	0.92	0.91	0.90
F1-Score	0.90	0.95	0.92	0.91	0.92
LR					
Precision	0.80	0.86	0.81	0.84	0.80
F1-Score	0.75	0.89	0.84	0.77	0.82
RF					
Precision	0.96	0.96	0.96	0.92	0.95
F1-Score	0.94	0.97	0.95	0.93	0.95

**Figure 7:**
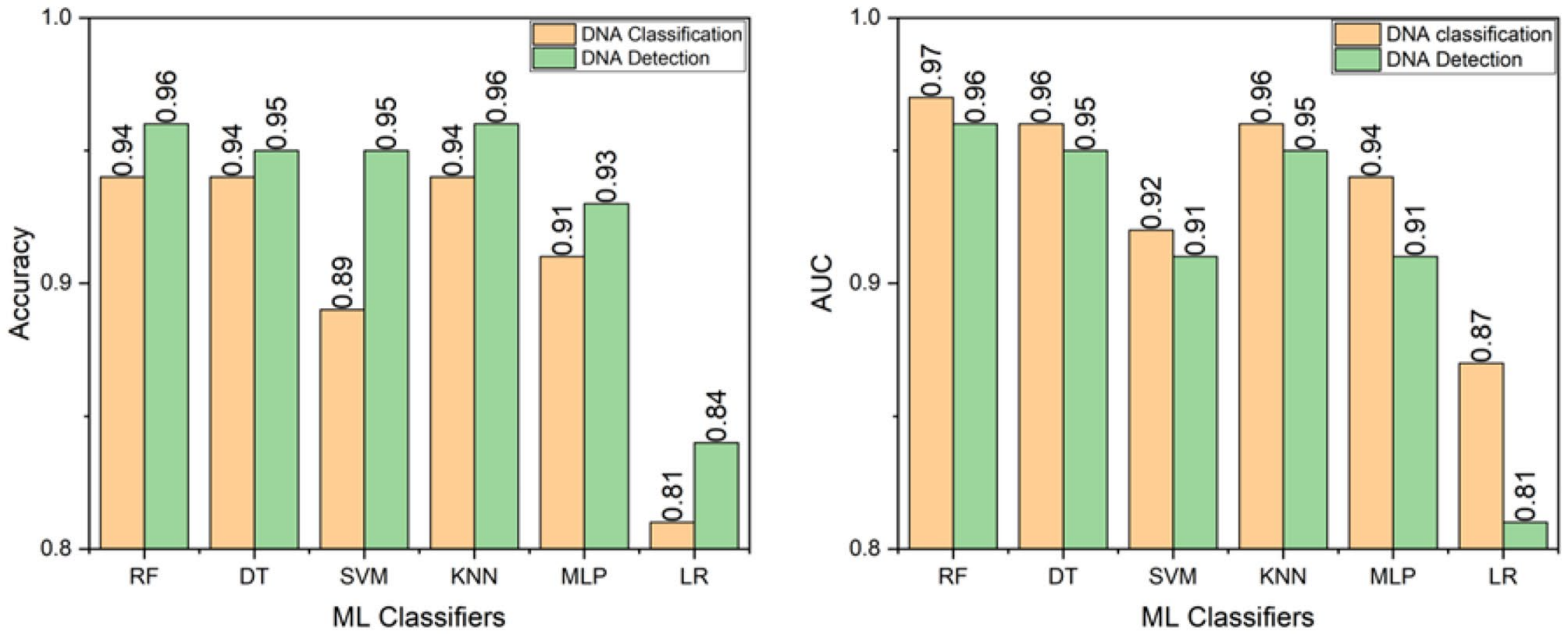
Accuracy (left) and AUC scores (right) of different classifiers and models.

According to the result of AUC, RF performed best as 0.97 overtaking DT and KNN, both of them are staying at 0.96, worst performance was by LR with 0.87. If we have a look at precision score, we also can say RF performs best among all the models as well as all the stages, which is reflected from Fig. 7. From the performance indicators, RF performs best among all the models.

**Figure 8:**
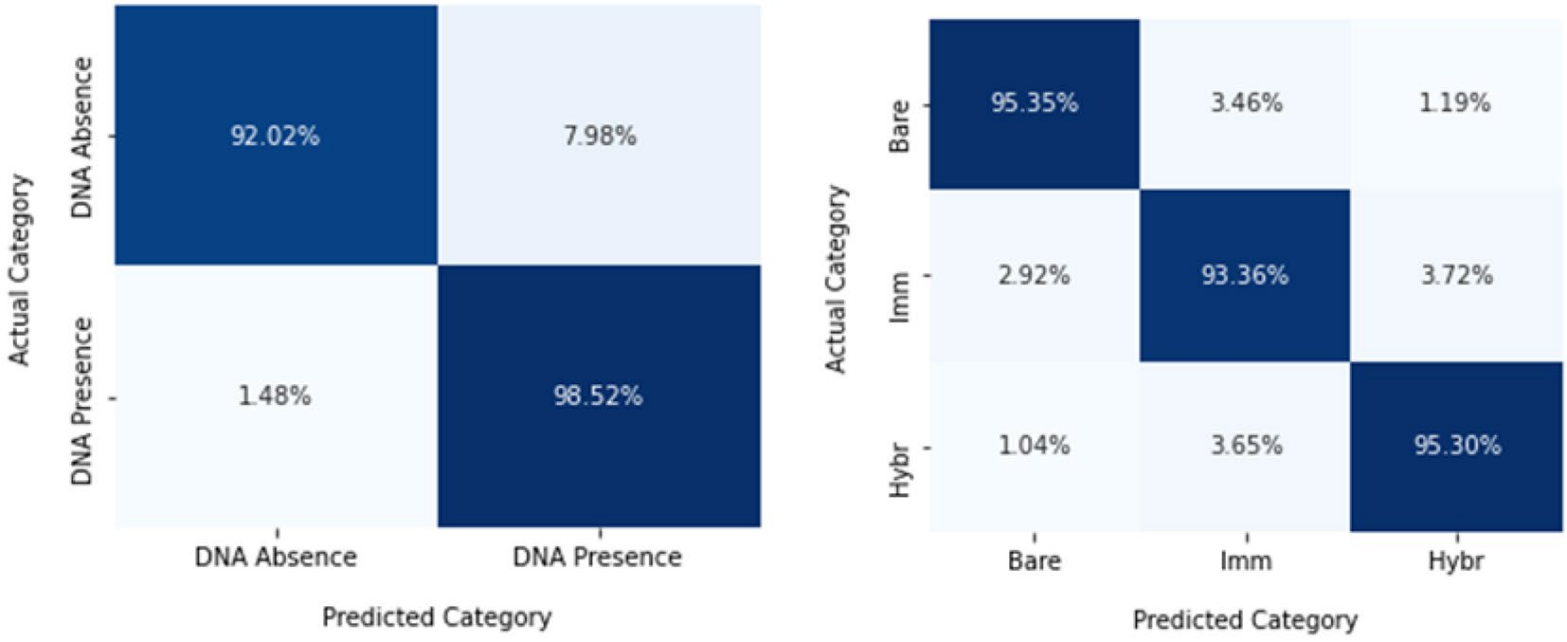
Normalized confusion matrix of RF classifier, DNA detection (Left) and DNA classification (right).

Normalized Confusion matrix (Fig. 8) for RF was generated to observe the true and false positives more closely for both classification and detection models. To evaluate the performance of best performing RF model, normalized confusion matrix is plotted for presenting effectiveness of the model. Normalization was done row-wise. Through this tabular summary of the number, it shows the correct and incorrect predictions made by RF classifier. Here, for DNA detection model, 98.52% true positive score was achieved for DNA presence, whereas DNA absence showed 92.02% score. It is also evident that, there was a 7.98% false positive score for DNA absence, while for DNA presence, the RF model achieved only 1.48% indicating RF performed well for DNA presence classification, compared to DNA absence.

On the other hand, for DNA classification models, normalised scores of 95.35%, 93.36% and 95.30% were achieved for detecting bare, immobilization and hybridization stages. The detecting performance of bare and hybridization was much higher compared to the immobilization stage.

### Hyper parameter optimization

**Figure 9:**
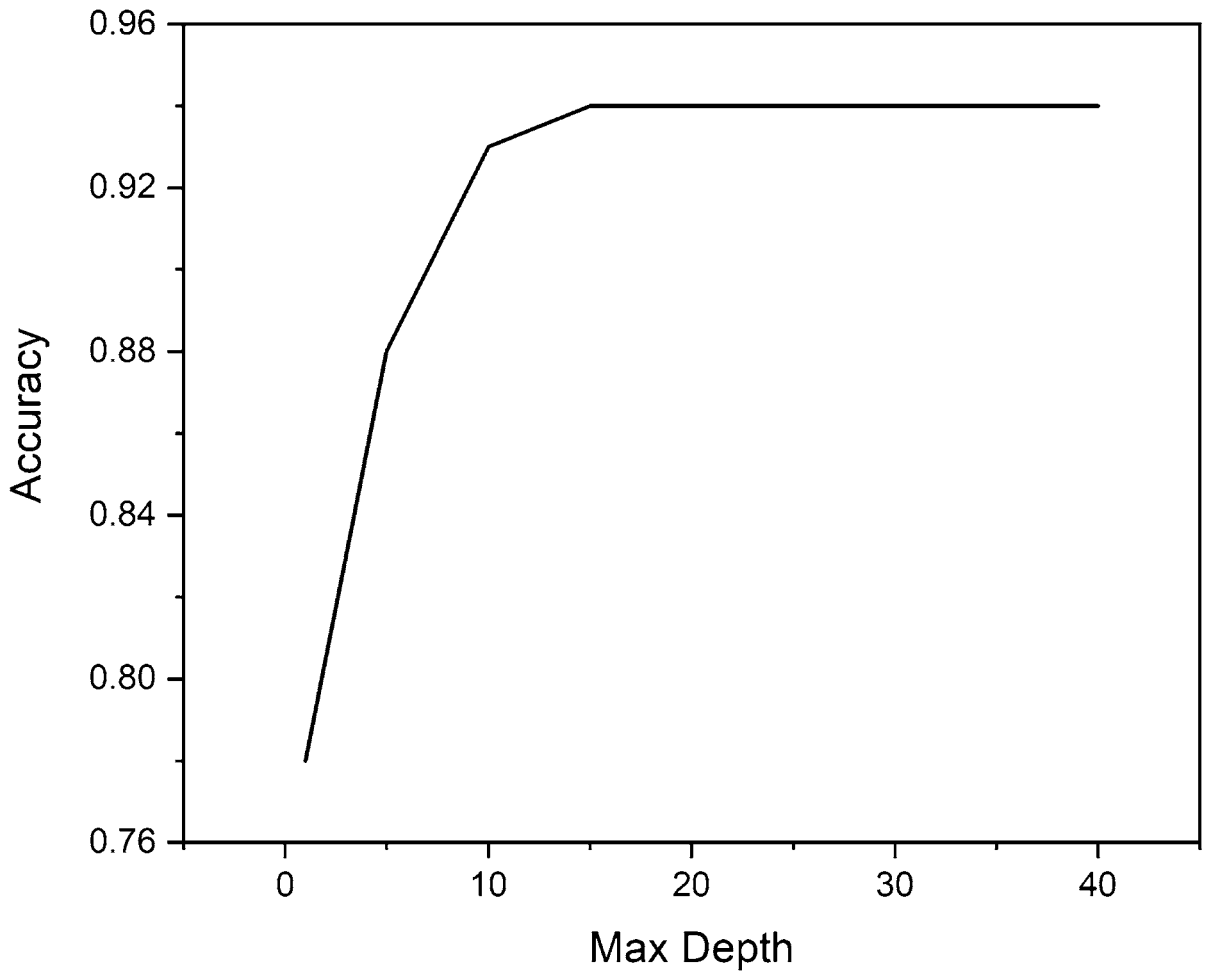
Effect of Max depth on RF classifier.

For better development of RF model, we have optimized the hyper-parameters for the RF model. After experimenting with different parameters, we found that, the RF model accuracy for current dataset is dependent on max depth parameter of the classifier. Our experiments shows, the accuracy sharply increases with max depth and after 15, the accuracy remains stable. The tuning result is presented in Fig. 9.

## Discussion

Current study explores different ML models for DNA classification and detection tasks. With the best of our knowledge this is the first study of its kind, which explored the applicability of ML models on SPR-based dataset for DNA classification and detection problems. While the lack of a similar model hinders us to compare our model performance, the current study set up a baseline to incorporate and consider sophisticated ML-models in the field of biosensor development.

Our statistical analysis found low variance within reflective angle classifiers, which made our task difficult to fit in a suitable model. Data pre-processing and t-SNE based feature extraction methods improved the classifiers’ classification capabilities. Often ML models, build upon low-variances dataset, suffer from low features, which subsequently results in high-biasness and overfitting problems in model performance^31^. Different data-processing and normalisation techniques can be helpful to address these issues, which allow for building robust and replicable models. Current research overcomes this low variance problem by clustering the dataset based on DNA attachment and DNA types, which not only increase the variances but also allows the scope to use the SPR data for different classification problems. For feature engineering, we tried different extraction methods, and t-SNE based feature extraction method is best suited for the current problem. Such t-SNE feature extraction worked well for other problems as well^32^.

We have divided the dataset, in terms of DNA presence and absence, and further extended it for the DNA type classification model. Overall, we found our detection model performs better than the classification model in terms of accuracy, precision and F1-scores. This result was expected from Figs. 5 and 6. DNA detection classifiers varied widely and showed wider t-SNE distribution, compared to DNA classification classifiers. In the case of DNA classification model, average angles for immobilization and hybridization stages showed almost similar patterns across the different gold thickness, as a result, precision and F1-scores for immobilization and hybridization stages scored lower compared to the bare classifier, which hampers overall accuracy of the models.

Further, we have evaluated different ML models, and found RF’s superior performance compared to DT, SVM, KNN, MLP and LR. RF has been widely utilised in numerous dataset and showed well performances in terms of diabetes patient detection^33^, heart disease detection^34,35^, liver disease detection^36^ and Parkinson’s disease detection^37^. A combination of LR and RF models gave the highest score for the prognosis of type-II diabetes. Models’ performance highly depends on several factors, e.g.- dataset, dataset preprocessing, feature extraction methods etc. and there is no generalized model to be fitted for every dataset. For example, MLP showed superior performance compared to SVM, LR, DT models for insomnia prediction^38^, While GP-based classifier with radial basis kernel (RBF) performed better than several ML models, including neural network, for diabetes prediction^39^. For current dataset, we incrementally tested numerous ML models and found RF and LR are the best and worst performers respectively. The probable reason can be due to the complex tree-based architecture of RF model, rather than the simple linear model of LR, which leverages RF to work on sophisticated classifiers and a plethora of hyper-parameters. RF uses multiple decision trees to capture non-linear relationships in high-dimensional and imbalanced data, which allows this model to maintain good accuracies^40^. We also couldn't rule out the performances of other models, especially DT and KNN, which showed consistent performances across different models. For different bio-medical and biosensor-based datasets, DT showed over 0.94 classification accuracies^41,42^. So, further study and hyper-parameter optimization are required for both DT and KNN models. Further, after testing different models, we experimented with different hyper-parameters of RF model. We found max-depth parameter is crucial for overall performance, and subsequently adjusted it to get the best output. Hyper-parameter optimisation is often an obvious choice for ML-models to test and customise for different dataset^43^.

Overall, all of the models, except LR, got accuracies above 0.89, with true positives and negatives above 0.92. It reflects the SPR data used in the current study is sufficiently large enough to train the models and our feature extraction method works well to differentiate each class. The high accuracy also gives an indication that average reflective angle data can be applicable for both DNA classification and detection problems for future predictive modelling. The reflective angles depend on numerous internal and external factors, e.g. gold thickness, permittivity and permeability, which needed to be considered for future modelling.

There are a few limitations of the current study. While t-SNE based feature extraction methods worked well for DNA detection model, our future task urge using other feature extraction methods to get higher scores for DNA classification models as well. Moreover, the size of the current dataset restricted us using only shallow, classical ML models, whereas convolution neural network-based models are an obvious choice for future model development. Also, hyperparameter tuning of the tested models, such as DT, KNN and MLP might give more promising outcomes. Finally, due to lack of SPR-based dataset, we cannot test our model for its wider applicability. So, testing similar model architectures and feature selection models will be utilized on future SPR-based biosensor studies.

## Conclusion

In order to rank classifiers and feature subsets, we explored SPR-based dataset, using various statistical, machine learning, and visualisation techniques. Based on the available resources, we proposed two innovative models, namely DNA detection and classification models with a view to utilising reflective angles and associated features for high-throughput biosensor development. Our step-by-step experiments of ablation studies, t-SNE net based feature extraction, evaluation of different ML classifiers and proposed RF-based model showed applicability and potentiality of ML models in the field of optical biosensors. Although few limitations still exists, current study set up a baseline for future ML studies in the field of therapeutics and biomedical diagnostics.

## Data Availability

The datasets used and/or analysed during the current study available from the corresponding author on reasonable request.
